# A Sub-Picoampere Measurement Algorithm for Use in Dosimetry of Time-Varying Radiation Fields

**DOI:** 10.3390/s24062012

**Published:** 2024-03-21

**Authors:** Michał Kuć, Maciej Maciak, Piotr Tulik

**Affiliations:** 1 Radiological Metrology and Biomedical Physics Division, National Centre for Nuclear Research, Andrzeja Sołtana 7, 05-400 Otwock, Poland; maciej.maciak@ncbj.gov.pl; 2 Institute of Metrology and Biomedical Engineering, Faculty of Mechatronics, Warsaw University of Technology, Św. A. Boboli 8, 02-525 Warsaw, Poland; piotr.tulik@pw.edu.pl

**Keywords:** radiation protection, recombination chamber, dosimetry, mixed radiation field, electrometry

## Abstract

Dosimetry based on gas detectors operating in the recombination and saturation region provides unique research opportunities but requires high-quality electrometers with a measuring range below 1 pA (10^−12^ A). The standard approach in electrometry is to strive to increase the accuracy and precision of the measurement, ignoring the importance of its duration. The article presents an algorithm for the measurement of low current values (from 100 fA) that allows both a fast measurement (with a step of 2.3 ms) and high accuracy (measurement error below 0.1%), depending on the measurement conditions and the expected results. A series of tests and validations of the algorithm were carried out in a measurement system with a Keithley 6517B electrometer and a REM-2 recombination chamber under conditions of constant and time-varying radiation fields. The result of the work is a set of parameters that allow for the optimisation of the operation of the algorithm, maximising the quality of the measurements according to needs and the expected results. The algorithm can be used in low current measurement systems, e.g., for dosimetry of mixed radiation fields using recombination methods and chambers.

## 1. Introduction

The technique of measuring low current values (above 1 fA), called electrometry, is an unpopular field of electronics, especially when the source of the measured signal is a device with a large volume (in the order of 1–10 dm^3^), electrical capacitance in the order of 100 pF and is sensitive to environmental conditions and vibration. This paper presents a description and a validation of a new low current measurement algorithm for use in gas detector-based dosimetry, using the REM-2 type recombination chamber as an example [1].

The standard measurement algorithm for ionizing radiation detectors described in this paper has inherent features that prevent or limit its applicability to the study of real radiation fields, such as time-varying fields. The innovation of the approach is based on the non-standard use of the functions of a Keithley 6517B-type electrometer [2], which reduces the previous problems associated with the sensitivity of the system to external conditions and significantly reduces the duration of the measurement. This paper describes and tests the new measurement algorithm using a REM-2 type recombination chamber in constant and time-varying radiation fields. Comparative measurements of the response of the existing and new measurement algorithms to a step change in the radiation field have also been carried out. The aim of this study is to prove that the measurement of the ionization current of the chamber by frequent measurement of the charge increment has a greater application advantage than the current mode measurement.

## 2. Materials and Methods

### 2.1. Measurement System

#### 2.1.1. Recombination Chamber Type REM-2

In the 1980s at the Institute for Nuclear Research (now the NCBJ National Centre for Nuclear Research), the most popular recombination chamber to date, the REM-2 was developed for radiation protection in workplaces where mixed radiation is present. A recombination chamber is a type of gas radiation detector that operates in the initial ion recombination voltage range. Dosimetry based on local ion recombination is not currently being developed in research institutes other than NCBJ. There are studies in the literature that refer to the occurrence of the local recombination of ions in the gas during the detection of ionizing radiation, but it is treated only as a phenomenon that worsens the measurement properties of the detectors [3,4].

The REM-2 recombination chamber, by virtue of to its design (large volume) and construction materials (tissue equivalent), is designed to measure the absorbed dose rate at a depth of 10 mm $D^{*}(10)$, the ambient dose equivalent at a depth of 10 mm $H^{*}(10)$ and the ambient radiation quality factor of mixed radiation fields $Q^{*}(10)$ [5,6]. Such chambers are designed for environmental dosimetry measurements for radiation protection in radiation fields of unknown compositions and energy spectrums. They have been successfully used to study radiation fields produced by isotope sources, nuclear reactors [7] and research and medical accelerators [8,9]; radiation fields with high particle energies [10,11,12]; environmental measurements [1]; and neutron monoenergetic fields [6].

The REM-2 chamber consists of two sets of flat ionization chambers made of tissue-equivalent material. The whole is placed in a single cylindrical container made of aluminum. The container is closed on both sides with steel lids. The design parameters and sensitivity of the REM-2 chamber are given in Table 1.

In this paper, an REM-2 type chamber with the serial number 8 was used. The standard designation of the detector is REM-2 no. 8.

The REM-2 chamber is a highly sensitive radiation detector, which in laboratory conditions allows for the study of radiation fields with intensities ranging from the natural background level to values approximately $10^{6}$ times higher. For a radiation field for which the detector records a radiation dose rate of the average value of the background level in Poland of about 250 nGy/h, an ionization current of $I_{S}=21.6\cdot 10^{-15}\;A$ (21.6 fA) is generated in the detector material. Measuring currents of this magnitude is problematic and requires sophisticated equipment and experimenter skills. The upper limit of the ionization current of the REM-2 detector reaches values of $10^{-6}\;A$ (1 μA).

#### 2.1.2. Dosimetry Based on Recombination Chambers

**Figure 1:**
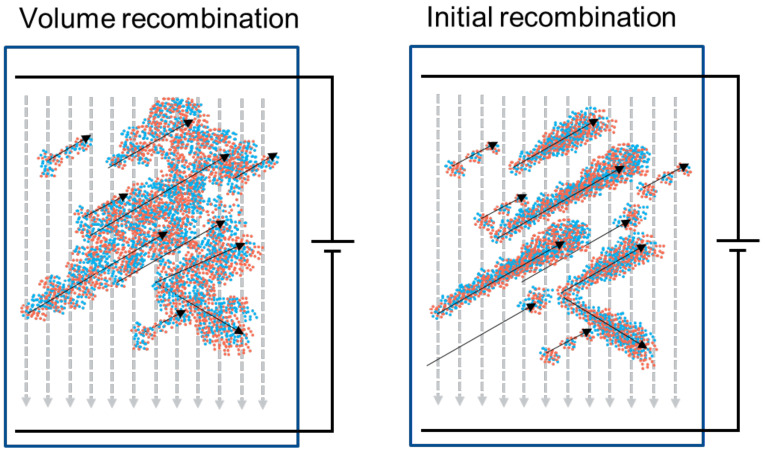
Comparison of the phenomenon of volume (**left**) and initial (**right**) recombination of ions in gas. The arrows represent the particle tracks. Initial recombination of ions occurs only between ions separated by the interaction of the same incident particle and does not depend on the number of particles incident on the detector (the clouds of ions formed as a result of the interaction of different incident particles do not overlap). Volumetric recombination also occurs between ions separated by the interaction of different particles and depends on the number of particles incident on the detector. The intensity of recombination occurrence, depending on its type, can be modified by the gas type and pressure inside the detector and the intensity of the electric field.

When exposed to radiation, the gas undergoes ionization, which generates a measurable ionization current, and recombination, which causes an irreversible loss of the generated charge. Two main types of recombination can be distinguished: initial (local) recombination and volume recombination. The former occurs only between ions separated by the interaction of the same incident particle and does not depend on the number of particles incident on the detector. Volume recombination, on the other hand, occurs between ions produced by the interaction of different particles and depends on the number of incident particles (Figure 1).

The contribution of recombination occurrence, depending on its type, is determined at the detector design stage and depends mainly on the working gas type and pressure and the range of electric field strength at the operating points. The study of recombination occurrence involves changing the value of the voltage applied to the detector, thus changing the drift time of the electrons to the electrode. Dosimetry based on the ion recombination in a gas uses, in addition to ion generation, the process of local recombination as a desired phenomenon that provides information about the unknown radiation field [13]. The main assumption in recombination methods is the dependence of the intensity of the occurrence of local ion recombination on the local ionization density of the gas and thus on the energy and type of radiation. Relating these quantities for a tissue-equivalent detector allows recombination methods and chambers to be used in mixed radiation fields (at least two types of radiation occurring simultaneously, most commonly neutrons and gamma), providing a link to the biological effects of radiation interactions [6,14,15].

Measurement methods for dosimetry based on ion recombination in the gas involve measuring the ionization current $I_{S}$ for the saturation voltage $U_{S}$ and comparing this value with the current for other detector-specific voltages, in particular the recombination voltage $U_{R}$. Saturation voltage is the voltage at which the charge collection efficiency is close to 100% (the electron drift time is so short that recombination in the gas does not occur to any significant degree).

Measurement under saturation conditions allows the determination of the total charge released in the detector due to the interaction with radiation. Depending on the calibration method and detector design, it is possible to determine a number of dosimetric quantities, including the absorbed dose D, the absorbed dose in water $D_{w}$, the kerma in air $K_{air}$, the tissue kerma $K_{tc}$, and the ambient absorbed dose at 10 mm depth $D^{*}(10)$. All the listed quantities can also be measured as rates of those quantities, i.e., their instantaneous values [16,17].

The recombination index of radiation quality $Q_{4}$ is a basic quantity determined using recombination dosimetry methods estimating the radiation quality factor Q [18]. For the certain voltages $U_{S}$ and $U_{R}$ specific for each recombination chamber type, the following relationship occurs:

(1) $$Q_{4}=\frac{1-\frac{I_{R}}{I_{s}}}{0.04}$$

where $Q_{4}\approx Q$, $I_{S}=I\left(U_{S}\right)$, $I_{R}=I(U_{R})$ [6].

A number of other dosimetry methods using local recombination of the ions in the gas have been developed, all of which are based on the comparison of current values for different detector operating voltages; in particular, the Recombination Microdosimetric Method (RMM) [15] measures the full current-voltage characteristics of the detector in the radiation field under test. RMM allows for determining the absorbed dose distribution according to restricted LET. The method requires current measurements for at least 50 different values of the detector polarization voltage.

#### 2.1.3. Electrometric Measurements

When considering methods for measuring the ionization current of ionization chambers, the most common concern is the accuracy of its determination, leaving aside the issues of frequency and the time resolution of the measurement. There are publications in the literature proposing the use of universal electrometers [1,8,19] as well as modified or new dedicated systems [20,21,22]. However, no work is available that analyzes the time-dependent measurement capabilities of dosimetric systems based on gas detectors. The major problems of dosimetric measurement systems based on recombination chambers are the long measurement duration, the required stability of the measured radiation field and the high sensitivity to variable slow (temperature, humidity) and fast (shock, vibration) environmental conditions [1]. The impact of each of these factors can be minimized by reducing the measurement time.

The realization of the measurement of the ionization current of the recombination chamber using an electrometer of the Keithley 6517B type [2] is possible based on two modes of operation of the device: current and charge (integrator) systems [23]. A DC amplifier, often an operational amplifier, is used to realize the input stage of the measuring system regardless of the mode of operation. The difference between the two cases is the use of a resistor or capacitor in the feedback loop of the operational amplifier. In the first case, the measured ionization current is proportional to the voltage at the output of the amplifier with a proportionality factor of the inverse of the value of the feedback loop resistor *R*. The time constant telling the stabilization of the system depends on the resistance *R* and the input capacitance of the system $C_{in}$.

(2) $$I=\frac{U_{out}}{R}(1-e^{-\frac{t}{RC_{in}}})$$

In the case of a charge circuit, the measured ionization current can be calculated as the derivative of the charge stored on the feedback loop capacitor *C* or as the derivative of the voltage at the amplifier output with a coefficient related to the capacitance of the feedback loop capacitor *C*.

(3) $$I=C\frac{dU}{dt}=\frac{dQ}{dt}$$

The wide range of measured ionization currents requires the electrometer system to be able to switch the measuring range. In both operating modes of the electrometer, this is realized by selecting the value of the feedback loop element, i.e., the resistance of the resistor *R* or the capacitance of the capacitor *C* [24].

**Figure 2:**
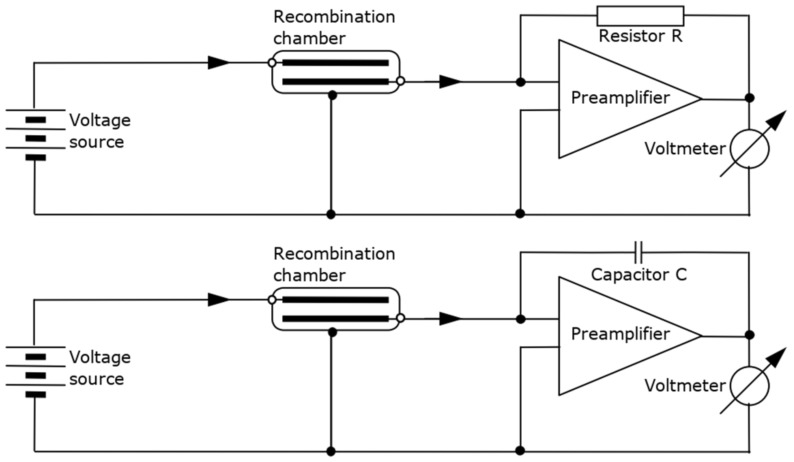
Simplified diagram of the measurement system built with a recombination chamber and a Keithley 6517B electrometer equipped with a high-voltage power supply and an electrometer circuit operating in current mode (**top**) or charge mode (**bottom**).

Simplified schematic diagrams of the measuring system built with a recombination chamber, a high voltage source and an electrometer circuit are shown in Figure 2. The upper diagram shows the electrometer operating in current mode, the lower diagram shows the charge mode.

There are many advantages of the charge mode over the current mode [2]. Among others, some advantages are as follows:

- Low current noise: A feedback resistor in current mode generates thermal noise. In charge mode, this resistor is replaced by a capacitor, whose thermal noise is significantly lower. This difference is evident at low currents on the order of fA.
- Shorter time to setpoint: The speed of the current circuit is limited by the time constant of the feedback loop. For example, with a feedback resistance R=1 GΩ and a REM-2 type recombination chamber capacitance $C_{in}=550\;pF$, the time constant $\tau =RC_{in}$ is 0.55 s. Unlike the current circuit, the charge circuit will respond immediately, and the response time is limited only by the speed of the operational amplifier.
- Random spurious pulses in the signal can be eliminated by averaging. The average charge carried per unit time of the random pulse trains can be measured as the final charge value. The average current value can then be expressed as the total charge divided by the total measurement time. This feature is particularly useful for averaging very small, transient currents.
- The individual pulses of interest are included in the measured signal. Due to the integral nature of the quantity being measured, any change is included in the signal. In current mode, individual pulses may be ignored due to the sampling rate.
- Single unwanted pulses can be removed from the signal. The differential charge-time waveform allows the current waveform to be determined with the advantages of the integrator circuit including fast circuit response.

#### 2.1.4. Keithley 6517B Type Electrometer

The Keithley 6517B type electrometer is a popular instrument for measuring very low currents. It has a built-in adjustable high-voltage power supply circuit with an operating range of ±1000 V. The instrument can be controlled manually via its front panel keys and remotely via a GPIB or RS-232 connection using SCPI commands. The electrometric circuit and the high voltage power supply allow for the complete electrical operation of a dosimetric measurement system based on a recombination chamber. Among its functionalities, several are important due to the developed algorithm:

- NPLC (Number of Power Line Cycle, PLC) parameter—the duration of the measurement of a single measurement point which depends on the multiplicity of the number of power line cycles. Significantly, it directly affects the duration and accuracy of the measurement.
- Digital signal filters: optional averaging and median.
- Synchronization of the measurement with the power line.
- Internal buffer for the collection of measurement data with a modifiable set of measured quantities, in particular those containing the measurement time and the measured value. The buffer capacity is 50,000 points. The maximum measuring frequency and simultaneous writing rate to the buffer is 425 rdg/s for the electrometer settings shown in Table 2.
- The signal noise suppression function applies only when the instrument is operating in current mode. The function increases the response time of the electrometer by low-pass filtering the signal.

In addition to the functions listed above, the electrometer offers an additional noise reduction function available only in current and resistance measurement modes. High capacitance at the electrometer input increases the reading noise. Enabling damping reduces this type of noise. However, damping also slows down the response of the measurement.

**Figure 3:**
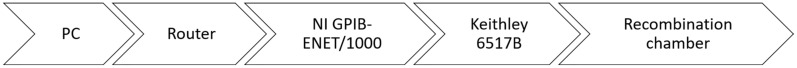
Diagram of the measurement system based on a recombination chamber and a Keithley 6517B type electrometer.

Figure 3 shows a diagram of the measurement system based on a recombination chamber and a Keithley 6517B electrometer. The recombination chamber is connected to the electrometer by a 15 m triaxial cable type HUBER+SUHNER G 02330 HT (Herisau, Switzerland) with the connector type HUBER+SUHNER 11 BNT-50-2-1/103 NE.

### 2.2. Measurement Algorithm

Dosimetric measurements carried out with recombination chambers involve the realization of a sequence of current (ionization current) measurements for different polarization voltages. The measurement is repetitive in nature, and each successive realization is independent of the previous one.

The existing measurement algorithm is based on a current measurement with an electrometer operating in current mode. The measurement data acquisition is performed point-by-point by reading the current measurement value with the fetch command. The final value of the current for a given voltage is taken as the average value of the current over time $I=\bar{I(t)}$. The maximum frequency of measuring the points of the vector I(t) in this mode on the existing control software is about 2 rdg/s. This is determined by the method of communication with the device and the frequency of updating this value in the electrometer (for the applied filter settings, NPLC and other significant parameters).

The new measurement algorithm is based on the most frequent charge measurement with the accurate timing of each measurement point (the accuracy of measurement point determination is ±1 μs). Measurement points are collected in the electrometer’s buffer, and after collecting the set number of points, all values are sent to the control computer. The trace:feed:control next command is used as the measurement execution command, which starts the predefined recording of the measurement data. Data processing to determine the current intensity is performed on the control computer and consists in particular of determining the differences in the charge vector over time $I\left(t\right)=\frac{\Delta Q}{\Delta t}$ and then determining the value of the current intensity estimator (by default as the mean value of the waveform).

Before starting the measurement, a number of parameters related to the measurement conditions, such as radiation intensity and stability, must be established: measurement range, NPLC value and number of buffer points. A fixed procedure for the selection of the above parameters has not been developed due to the excessive number of variables in the measurement conditions (stability of the radiation field, stability of the environmental conditions and values of the measured current intensity) and to the different expected measurement results (monitoring of the radiation field, measurement of the radiation dose rate over time, study of the variation of the radiation field, measurement of a stable radiation field set to a low measurement error, study of pulsed fields).

**Figure 4:**
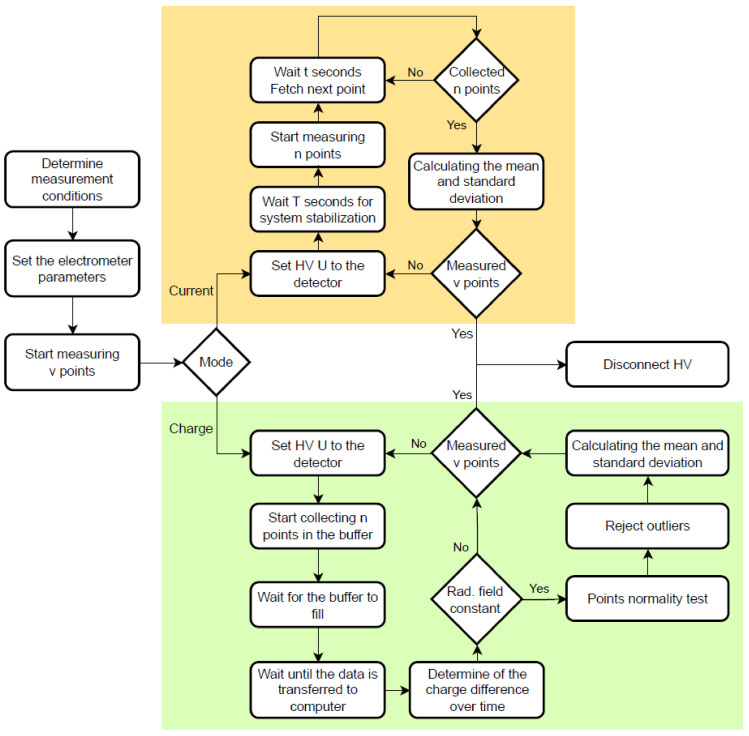
A flowchart of the existing (orange) and the new (green) measurement algorithms. The approximate measurement times for a current of about 100 pA (approximate calibration conditions of the REM-2 type detector in the NCBJ laboratory $D^{*}(10)\approx 1.16\;mGy/h$) are for existing and new algorithms and are 220 s and 10 s, respectively. The existing measurement algorithm is based on the electrometer current mode and data acquisition by fetch command. The new algorithm uses a charge mode and data acquisition collected in an internal buffer using the trac:feed:cont next command.

The standard duty cycle of both algorithms for measuring the current values for a single detector supply voltage is shown in Figure 4.

Due to the integer nature of the charge measurement, continuous measurement in this mode must not last longer than until the upper limit of the measurement range is reached or the electrometer buffer overflows. Table 3 shows the maximum duration of the continuous measurement in charge mode. Continuing the measurement after the upper limit of the measurement range is reached requires discharging the input circuit. The inability of the system to measure due to reaching the upper limit of the measurement range is approximately 1 s. The speed of data transfer from the electrometer to the GPIB bus control computer is a maximum of 3300 rdg/s.

### 2.3. K6517B and RecCham Computer Software

**Figure 5:**
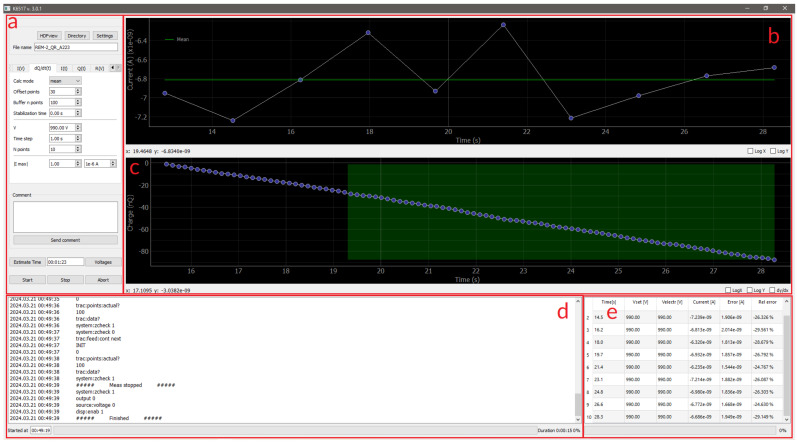
View of the software for performing measurements with SCPI-compliant instruments, in particular the Keithley 6517A and 6517B electrometers. The window is divided into five areas: (**a**) the area for setting the measurement mode, session parameters and measurement control; (**b**) the overall graph of the entire measurement; (**c**) the sub-graph for one measurement execution; (**d**) the communication flow window and (**e**) the measurement results window.

The K6517B v. 3.0.1 computer software was prepared to perform measurements with SCPI-compliant instruments, in particular the Keithley 6517A and 6517B electrometers. The software was tested and used to test a series of silicon photomultipliers [25]. The software allows connection to a compatible device via GPIB, TCP/IP or RS-232 and operation in six measurement modes based on both an internal buffer and reading the current measurement value (fetch) presented in Table 4:

The software was developed in the Python3 language using libraries including pyvisa, multiprocessing, PyQt5, h5py and numerical processing NumPy. Figure 5 shows a view of the software GUI. The window is divided into five areas: (a) the area for setting the measurement mode, session parameters and measurement control; (b) the overall graph of the entire measurement; (c) the sub-graph for one measurement execution; (d) the communication flow window and (e) the measurement results window.

The software is characterized by its flexibility in terms of measurement mode extension and simplicity in any configuration of the instrument operation. The settings related to the course of the measurement are set from the program window, while the settings of the electrometer related to the technical aspects of the implementation of the measurement can be modified using the configuration file.

The presented software was developed as a tool for laboratory measurements and for testing new measurement algorithms for dosimetry based on recombination chambers.

In parallel to the K6517 software, the RecCham measurement system was developed with its communication and measurement process part, while the whole system allows the performance of dosimetry measurements based on real multi-detector recombination dosimetry methods. The RecCham system allows radiation fields to be tested by pre-trained users. It allows the automation of the measurement process, starting with the self-loading of the detector calibration parameters, as well as the selection of the operating parameters to assess the quality of the results obtained. The system was also developed in the Python3 programming language, while its architecture allows integration with other independent systems and measurement systems, creating a larger, dedicated tool that realizes extensive, sequential measurement processes.

**Figure 6:**
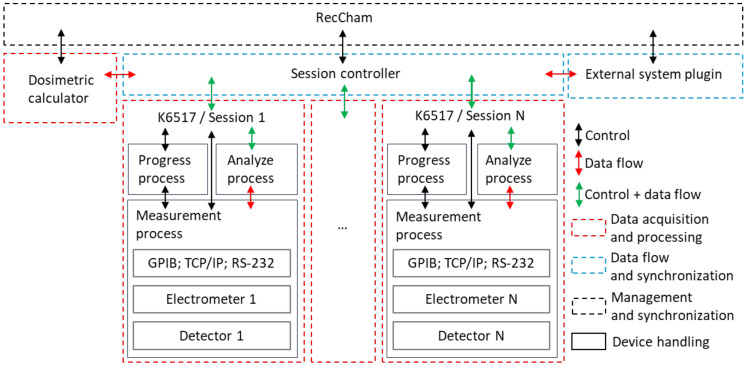
A block diagram of the RecCham measurement system. The nesting of the K6517 program within the RecCham system as a single session handling the measurement process of the Keithley 6517A/B device is presented. The concept of connecting an external system as a slave to RecCham is shown.

The K6517 and RecCham v. 22.08 software provide a number of opportunities to explore new measurement algorithms, create new dosimetry systems and, thanks to their versatility, support the activities of other laboratories. Figure 6 shows a block diagram of the RecCham measurement system. It can be seen that the K6517 program is nested within the RecCham system as a single session that handles the measurement process of the Keithley 6517A/B device. A diagram of the communication between the processes and the tasks performed by the different components of the system is shown. An external system plug-in is also presented, which must be compatible in terms of the communication protocol and control and the measurement data format.

The system has implemented the following dosimetry methods:

- twin-chamber measurement method for the gamma and neutron doses discrimination;
- monitored measurement of absorbed dose rate, recombination index of radiation quality and ambient dose equivalent and
- monitored measurement of dose distribution according to linear energy transfer in mixed radiation fields.

### 2.4. Experimental Sites

#### 2.4.1. Measurement of Ionization Current in Isotope Radiation Fields

Measurements in radiation fields from isotope sources were performed at the accredited (AP070 accreditation) calibration laboratory operated by the Laboratory for Dosimetry Measurements (LPD) at the National Center for Nuclear Research. This type of radiation field has been assumed to be time-constant, unlike the accelerator-based radiation sources described in the next paragraph. A Tema Sinergie IM6M automatic irradiator with a calibration bench and ^137^Cs and ^60^Co gamma-ray sources were used. The range of kerma rates in the air at the measurement points on the calibration bench was from 900 nGy/h to 9.35 mGy/h.

A series of measurements of the ionization current of the REM-2 chamber in the isotope gamma radiation fields of ^137^Cs and ^60^Co were performed. The behavior of the measurement system (REM-2 type recombination chamber, electrometer, control computer and K6517 software) operating with the new measurement algorithm was studied for nine different radiation levels and for 16 different electrometer operating settings. For each measurement point on the calibration bench, the reference current $I_{REF}$ was determined as the value of the ionization current in the chamber based on the known values of the ambient absorbed dose $D^{*}(10)$ and the calibration factor of the recombination chamber $A_{D^{*}\left(10\right)}$.

(4) $$I_{REF}=D^{*}\left(10\right)\cdot A_{D^{*}(10)}$$

The expanded uncertainty in the determination of the recombination chamber calibration factor according to the internal procedures of the accredited LPD calibration laboratory is ±5%.

Each measurement series contained a different number of measurement points depending on the value of the NPLC parameter (Table 5). The K6517 software worked in the mode of measuring the current over time by measuring the charge increase using the buffer $I(t)=\frac{\Delta Q}{\Delta t}(t)$. The average value of the current course $\bar{I}=\frac{\sum_{i=1}^{n}I_{i}}{n}$ was taken as the current value.

The performed measurements allowed us to obtain information about the statistical measurement error of the mean value of the ionization current measured with the new measurement algorithm. The standard error of the mean SE of the current $\bar{I}$ was used as an estimator of the measurement error [26]:

(5) $$SE=\sqrt{\frac{\sum_{i=1}^{n}{\left(I_{i}-\bar{I}\right)}^{2}}{N(N-1)}}$$

The standard error of the mean was presented as the relative value of the SE to the mean value of the current $\bar{I}$:

(6) $$SE_{REL}=\frac{SE}{\left|\bar{I}\right|}\left[\%\right]$$

Measurements were also made of the current values and the standard error of the mean current for the parameters of the series of measurements described above. The measurements were performed with a simplified measurement system without the detector section (current source, electrometer, control computer and K6517 software). A Keithley 261 analog current source with an output range of ±10^−14^ A to ±10^−4^ A was used as the signal source. The accuracy of the output current was ±(0.25% + 1 rdg) for the $10^{-7}$ range and ±2.0% for the $10^{-12}$ range. The minimum value of the current set on the Keithley 261 was 0.5 pA due to the accuracy of the instrument operation.

#### 2.4.2. Study of the Response of the Measurement System to a Step Change in the Ionization Current of the Recombination Chamber

A comparative measurement of the ionization current of the REM-2 type detector for an abrupt change in the radiation dose rate (measurement of the radiation dose rate during the opening and closing of the radiation beam) was performed for an electrometer operating in current mode and in charge mode. A ^137^Cs gamma source exposed from a Tema Sinergie IM6M automatic irradiator was used. The estimated exposure and retraction time of the source is 0.2 s. The measured ionization current was approximately 80 pA. A continuous measurement was performed using an internal buffer for the electrometer operating in current mode with a measuring range of 200 pA and in charge mode with a measuring range of 2 nC. The NPLC value was one in both cases.

#### 2.4.3. Measurement of the Detector Ionization Current in a Time-Varying Radiation Field

The Keithley 6517B electrometer provides charge measurement results with different resolutions depending on the fixed measurement time step (depending on the NPLC parameter shown in Table 5), gives the charge measurement results with different resolutions. The measurement resolution for NPLC parameters of 0.01, 0.1, 1 and 10 is expressed in terms of the number of full digits in the range of 0–9 N and the digits at the most significant position of 0 or 1 (denoted as (1/2)) and is 3 (1/2), 4 (1/2), 5 (1/2) and 6 (1/2), respectively. For example, a value of 3 (1/2) means that ±1999 values can be displayed, i.e., three full digits in the range of 0–9 and one digit, the most significant digit of 0 or 1) [2].

For such a notation, it is possible to determine the smallest measurement value $Q_{min}$ for a given range *r* with the number of full significant digits *N* expressed as a charge:

(7) $$Q_{min}=\frac{1}{2\cdot 10^{N}}\cdot r\;[C]$$

Taking into account the different resolutions of the display of the results and the different time step $t_{step}$, Table 6 shows the minimum values of the current $I_{min}$ for which the charge increase in the time step of the measurement is at least the minimum measured value for the given range. The values are determined as $I_{min}=\frac{Q_{min}}{t_{step}}$. The values do not indicate the minimum current values that can be measured with the given electrometer settings, but the values for which the accuracy of the charge time step measurement at each measurement point are determined with an error related to the resolution of the display of no more than 100%.

To date, dosimetry studies in pulsed radiation fields using recombination methods have been based on measuring the corresponding radiation dose by measuring the charge accumulated during the exposure (measurement time of at least 1 s due to limitations of the existing measurement algorithm). This approach did not provide the possibility of determining the instantaneous parameters of the radiation fields. The new measurement algorithm assumes the ability to measure radiation dose rate values during pulses of 10 ms or longer.

A series of measurements were performed in the X-ray beam environment to assess the feasibility of testing pulsed radiation fields using recombination chambers. The study made it possible to determine the accuracy of the response of the measurement system working with the new algorithm during very short radiation pulses. The variable parameter during the experiment was the exposure time $t_{exp}$ in the range from 10 ms to 250 ms (10 measurement repetitions were performed for each exposure time). The X-ray source used was a FLEXAVISION HB X-ray system from SHIMADZU at the Faculty of Mechatronics at the Warsaw University of Technology. The X-ray scanner was operated with an accelerating voltage of 70 kV and a current of 125 mA in the radiography mode and with an accelerating voltage of 70 kV in the fluoroscopy mode. The measurements were performed with a REM-2 no. 8 detector placed on the scanner table at a distance of 50 cm from the beam axis; the distance of the X-ray tube focus from the diagnostic couch was 1037 mm. To increase the sensitivity of the detector, a uniform PMMA phantom of 24 cm × 24 cm × 15 cm was centered at the beam axis on the diagnostic couch.

For each exposure repetition, the current value $I_{calc}$ was determined as the total charge accumulated in the electrometer during the pulse duration ($Q_{stop}-Q_{start}$) divided by the pulse duration $t_{exp}$ set on the instrument.

(8) $$I_{calc}=\frac{Q_{stop}-Q_{start}}{t_{exp}}$$

The average value of $I_{calc}$ from 10 repetitions for each pulse duration was used as the reference value for the measured current value $I_{meas}$.

The measured current value $I_{meas}$ was determined as the average value of the course of the charge derivative I(t) during the pulse.

(9) $$I\left(t\right)=\frac{\Delta Q}{\Delta t}\left(t\right)$$

(10) $$I_{meas}=\frac{\sum_{i=1}^{n}I_{i}}{n}$$

## 3. Results

### 3.1. Measurement of Ionization Current in Isotope Radiation Fields

Table 7 shows the relative standard error of the mean SE of the ionization current of the REM-2 recombination chamber in the gamma radiation field for different values of the ambient absorbed dose rate D*(10) and different electrometer settings. The error is calculated relative to the mean of the measurement points.

In order to study the statistical measurement errors of the measurement system without the influence of the detector system and radioactive sources, measurements similar to those described above were performed using a Keithley 261 current source (the previously mentioned measurement system without the detector part). The results of these measurements are given in Table 8.

### 3.2. Study of the Response of the Measurement System to a Step Change in the Ionization Current of the Recombination Chamber

**Figure 7:**
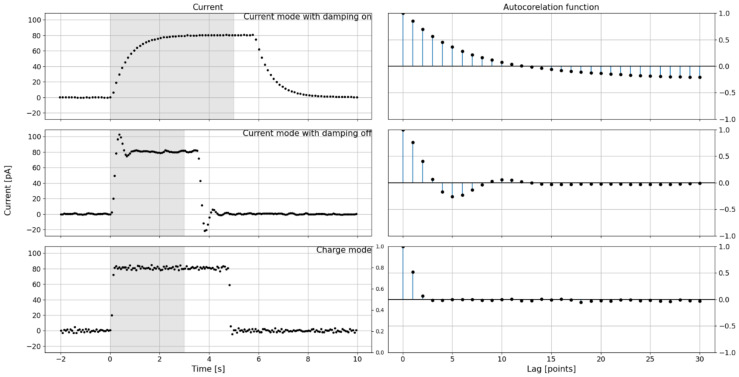
Measurements of the ionization current course of a REM-2 no. 8 chamber for step changes caused by exposure to the radiation source. Comparison of current waveform *I*(*t*) (left column of graphs) and autocorrelation functions (right column of graphs) of the ionization current for a Keithley 6517B-type electrometer operating in current mode with the damping function on (top row), with the damping function off (middle row) and in charge mode, for which the ionization current was determined as $I(t)=\frac{\Delta Q}{\Delta t}(t)$ (bottom row). The gray shading indicates the area for which autocorrelation functions were determined.

The ionization current waveforms were measured and autocorrelation functions of the ionization current of the REM-2 no. 8 chamber were determined for step changes in measurement conditions. The changes consisted of exposing and then concealing the ^137^Cs radiation source. Measurements were carried out for the three operating configurations of the measurement system; the results are shown in Figure 7:

- Top row: current mode with noise damping enabled, NPLC = 1, digital filters disabled, measurement range 200 pA.
- Middle row: current mode with noise damping disabled, NPLC = 1, digital filters disabled, 200 pA measurement range.
- Bottom row: charge mode, NPLC = 1, digital filters disabled, measurement range 2 nC, current determined as a charge increase.

The left column of the graphs contains the time courses; the right column of the graphs contains the autocorrelation functions for the shaded areas of the time courses. The graph shows the moments of exposure of the source from the irradiator and the moment of retraction of the source. The time to reach 10% deviation from the target stable signal value for the next three settings of the measurement system were determined with an uncertainty of ±0.1 s and are 1.5 s, 0.5 s and 0.1 s, respectively.

### 3.3. Measurement of Detector Ionization Current in a Time-Varying Radiation Field

An attempt was made to determine the ionization current of the chamber during the RTG accelerator pulse. Table 9 shows the values of the ionization current *I_calc_* and *I_meas_* of the REM-2 recombination chamber for different beam exposure times *t_exp_*. and the relative difference between the mean *I_meas_* (for 10 exposure repetitions) and *I_calc_* values. For NPLC values of 0.01 and 0.1, measurements for exposure times greater than 100 ms were not performed, because for these *t_exp_* the results are well-defined due to the long pulse duration relative to the measurement time step. The remaining empty points in the table are due to the impossibility of determining *I_meas_* values because the pulse duration is too short for a given measurement time step (for this reason, measurements were not performed for NPLC = 10, for which the measurement time step is 602 ms); for these points, at most one point gave a value higher than the background value, which was considered insufficient to correctly determine the ionization current *I_meas_*.

## 4. Discussion

### 4.1. Measurement of Ionization Current in a Time Constant Radiation Field

The presented results confirm the correctness of the presented algorithm for the determination of constant current values measured with a Keithley 6517B type electrometer. The method works both in a system with an REM-2 type detector and with a reference Keithley Model 261 Picoampere current source. The results show that the measurement error and the difference between the measured values of the current and the reference value increase as the measurement range increases and the NPLC value decreases. For both sets of measurements (in the gamma-ray field and with a current source) the relationship is identical. At higher NPLC values, the electrometer has higher measurement accuracy, so the SE is lower and the measured current value is closer to the reference value. The trend described above is common to both sets of measurements. The difference between the measured and reference current values for the range of current values below 1 pA is due to the error in the *I_REF_* current reference values. In the case of measuring the ionization current of the recombination chamber, the difference is related to the accuracy of determining the ambient dose at the measurement point, in the case of using a current source to the accuracy of the instrument, and in both cases to the systematic error of the charge measurement electrometer.

Carrying out the presented measurements provides an opportunity to correctly select the operating parameters of the measurement algorithm, i.e., the selection of the electrometer operating parameters and the duration of the measurement, in order to obtain the expected quality of results in terms of measurement error and time resolution. The measurements cover a wide range of current and, at the same time, a wide range of radiation field intensity, where the REM-2 type detector was used. The results allow the evaluation of the applicability of the new measurement algorithm depending on the measured values of the ionization current.

### 4.2. Study of the Response of the Measurement System to a Step Change in the Ionization Current of the Recombination Chamber

The system responses were compared to confirm the advantages of the charge mode over the current mode in terms of system response time. In both cases of system operation, the time responses of the current mode were significantly longer than those of the charge mode. In addition, the use of the built-in damping function in the charge mode, which smooths the course and significantly reduces the measurement error, significantly increases the system’s stabilization time.

When the system is operating in the current mode with the damping turned off, the waveform has an oscillatory character for a sudden change in the measured value. In addition, the waveform shows an overshoot when the measurement conditions change. The autocorrelation plots confirm the observation of the oscillatory nature of reaching the target value for the system operating in current mode with the damping off. The system operating in charge mode is significantly faster, and the speed of the system’s response time cannot be determined from the presented graphs due to the limitation associated with the rate of change of measurement conditions.

The comparison of measurement systems presented in this section proves that the current mode is not suitable for performing time-varying radiation field measurements for ionization currents less than 80 pA.

### 4.3. Measurement of Detector Ionization Current in a Time-Varying Radiation Field

The results of ionization current measurements obtained for a time-varying radiation field allow for the determination of the value of the measurement error depending on the time-varying parameters of the radiation field. For the points where the value could be determined, the difference between the value of $I_{calc}$ and $I_{mean}$ was maximum 3.3%. The results of the study allow to select the working parameters of the new measurement algorithm with respect to the studied radiation field.

The presented results of the analysis allow us to conclude that the REM-2 type recombination chamber, in combination with an electrometric system operating in charge mode, provides the possibility of studying pulsed radiation fields by measuring the radiation dose rate during a pulse of at least 10 ms duration. The approach presented in this paper opens new possibilities for the study of time-varying radiation fields.

## 5. Conclusions

This paper presents a new measurement algorithm as a new approach to the problem of low-current measurement that can replace existing electrometric methods in recombination chamber-based dosimetry. Through a series of experiments, it was shown that the new approach significantly extends the applicability of chamber-based dosimetry and recombination methods.

The paper presents the measurement results in tabular form, which can be used to adjust the operating parameters of the measurement system for both constant and time-varying radiation field conditions.

The results obtained in this paper confirm the feasibility of using a REM-2 type recombination chamber in a system with the developed measurement algorithm in pulsed radiation fields. The innovation demonstrated in the work is the possibility of using a charge-based measurement algorithm to study time-varying radiation fields with a minimum duration of 10 ms. At the same time, it was shown that the current mode of the Keithley 6517B electrometer has a significantly longer response time compared to the charge mode. The studies presented in this paper also provide a basis for the development of chamber-based dosimetry and recombination methods for use in measurement conditions not previously possible.

## Figures and Tables

**Table 1:** Parameters of the REM-2 no. 8 recombination chamber, data from the detector information brochure and calibration of the detector performed in an accredited laboratory using a ^137^Cs radiation source, reference quantity $D^{*}(10)$.

Chamber Identification	REM-2 No. 8
Chamber dimension	Ø 167 × 403 mm
Mass	6.5 kg
Active volume	1800 cm^3^
Gas type	95% CH_4_ + 5% N_2_
Gas Pressure	approx. 8 bar
Electrical capacity *C_in_*	550 ± 50 pF
Ambient dose rate range D*(10)	0.005 mGy/h ÷ 300 mGy/h
Ambient dose rate accuracy D*(10)	15%
Saturation voltage *U_S_*	990 V
Recombination voltage *U_R_*	60 V
Calibration factor A	(8.64 ± 0.43) × 10^−11^ A/(mGy/h)
Dark current	<1 × 10^−13^ A (<100 fA)
Calibration date	19 September 2022
Relative neutron sensitivity	1.03
Relative gamma sensitivity	1.00

**Table 2:** Keithley 6517B electrometer parameter settings to maximize measurement frequency.

Function	SCPI Command
NPLC set to 0.01	sense:function:mode:nplc 0.01 (mode: current or charge)
Digital filters disabled	sense:mode:average:state 0; sense:mode:median:state 0 (mode: current or charge)
Front panel of the device disabled	disp:enab 0
Relative humidity and temperature measurement disabled	system:tsc 0; system:hsc 0
Synchronization of measurement with mains power line disabled	system:lsync:state 0

**Table 3:** The maximum continuous measurement time as a function of the measured current value I_REF_, the NPLC value and the charge measurement range of the Keithley 6517B electrometer. The maximum time is also limited by the fixed charge measurement range and the capacity of the electrometer’s internal buffer of 50,000 points.

Continuous Measurement [s]										
Range [nC]	NPLC	Reference Current I_REF_ [A]								
		1 × 10^−13^	5 × 10^−13^	1 × 10^−12^	5 × 10^−12^	1 × 10^−11^	5 × 10^−11^	1 × 10^−10^	5 × 10^−10^	1 × 10^−9^
2	10	20,000	4000	2000	400	200	40	20	4	
	1	3100	3100	2000	400	200	40	20	4	2
	0.1	400	400	400	400	200	40	20	4	2
	0.01	100	100	100	100	100	40	20	4	2
20	10	30,100	30,100	20,000	4000	2000	400	200	40	20
	1	3100	3100	3100	3100	2000	400	200	40	20
	0.1	400	400	400	400	400	400	200	40	20
	0.01	100	100	100	100	100	100	100	40	20
200	10	30,100	30,100	30,100	30,100	20,000	4000	2000	400	200
	1	3100	3100	3100	3100	3100	3100	2000	400	200
	0.1	400	400	400	400	400	400	400	400	200
	0.01	100	100	100	100	100	100	100	100	100
2000	10	30,100	30,100	30,100	30,100	30,100	30,100	20,000	4000	2000
	1	3100	3100	3100	3100	3100	3100	3100	3100	2000
	0.1	400	400	400	400	400	400	400	400	400

**Table 4:** Description of the K6517 v. 3.0.1 software measurement modes.

	Mode Description	Measured Quantity	Electrometer Mode
1	Measurement of current-voltage characteristics by measuring the charge increase using the buffer	$I(V)=\frac{\Delta Q}{\Delta t}(V)$	Charge
2	Direct measurement of current-voltage characteristics	I(V)	Current
3	Measurement of current over time by measuring the charge increase using the buffer	$I(t)=\frac{\Delta Q}{\Delta t}(t)$	Charge
4	Direct measurement of current over time	I(t)	Current
5	Measurement of charge over time using the buffer	Q(t)	Charge
6	Direct measurement of resistance versus voltage	R(V)	Resistance

**Table 5:** Settings for the measurement of the ionization current in a time-constant radiation field using Keithley 6517B electrometer, the number of measurement points for each measurement series and the measurement time step depending on the value of the NPLC parameter.

NPLC	Buffer Time Step t [ms]	Buffer Points *N*
0.01	2.3 ± 0.1	1000
0.1	8.0 ± 0.1	1000
1	60.2 ± 0.1	500
10	602.0 ± 0.1	200

**Table 6:** Minimum current values for which the charge increase in the measurement time step is at least one significant digit.

NPLC	$\mathrm{Time}\;\mathrm{Step}\;t_{step}$ [ms]	Resolution [*N* (1/2)]	Range r [nC]			
			2	20	200	2000
0.01	2.3	3.5	4.35 × 10^−10^	4.35 × 10^−9^	4.35 × 10^−8^	4.35 × 10^−7^
0.1	8	4.5	1.25 × 10^−10^	1.25 × 10^−9^	1.25 × 10^−8^	1.25 × 10^−7^
1	60.2	5.5	1.66 × 10^−11^	1.66 × 10^−10^	1.66 × 10^−9^	1.66 × 10^−8^
10	602	6.5	1.66 × 10^−12^	1.66 × 10^−11^	1.66 × 10^−10^	1.66 × 10^−9^

**Table 7:** Relative standard error of the mean $SE_{REL}$ ionization current of the REM-2 no. 8 recombination chamber in the gamma radiation field for different radiation dose rate power values and different electrometer settings (NPLC and measurement range). The error is calculated relative to the sample average of the measured values. Measurements were performed in LPD using isotope radiation fields of ^137^Cs and ^60^Co.

Range [nC]	NPLC	Reference Current *I_REF_* [A]								
		1.00 × 10^−13^	5.00 × 10^−13^	1.00 × 10^−12^	5.00 × 10^−12^	1.00 × 10^−11^	5.00 × 10^−11^	1.00 × 10^−10^	5.00 × 10^−10^	1.00 × 10^−9^
		Reference Ambient Dose Equivalent D*(10) [μGy/h]								
		0.93	4.67	9.35	46.73	93.46	467.29	934.58	4672.90	9345.79
2	10	13.24%	2.34%	1.08%	0.26%	0.13%	0.03%	0.02%	0.02%	
	1	83.57%	9.06%	4.65%	0.93%	0.50%	0.11%	0.06%	0.02%	0.03%
	0.1	164.35%	23.44%	11.40%	2.35%	1.16%	0.23%	0.12%	0.03%	0.04%
	0.01		52.99%	28.25%	6.30%	3.14%	0.64%	0.32%	0.09%	0.09%
20	10	12.16%	2.27%	1.21%	0.22%	0.13%	0.03%	0.02%	0.01%	0.01%
	1	179.42%	10.67%	4.61%	1.05%	0.49%	0.11%	0.05%	0.02%	0.01%
	0.1	232.37%	52.98%	19.75%	5.38%	2.95%	0.46%	0.24%	0.05%	0.03%
	0.01						5.43%	2.90%	0.24%	0.12%
200	10	7.12%	2.19%	1.11%	0.26%	0.13%	0.04%	0.02%	0.01%	
	1	72.78%	19.73%	13.20%	3.86%	1.71%	0.28%	0.16%	0.04%	0.02%
	0.1						5.43%	2.87%		0.10%
	0.01								5.29%	2.87%
2000	10	38.89%	8.45%	4.75%	1.11%	0.57%	0.12%	0.06%	0.01%	0.01%
	1					13.89%	4.28%	1.66%	0.18%	0.09%
	0.1								5.28%	2.87%

**Table 8:** Relative standard error of the mean current $SE_{REL}$ measured with a Keithley 261 current source for different electrometer settings (NPLC and measuring range) The error is calculated relative to an average of the measured values.

Range [nC]	NPLC	Reference Current *I_REF_* [A]							
		5.00 × 10^−13^	1.00 × 10^−12^	5.00 × 10^−12^	1.00 × 10^−11^	5.00 × 10^−11^	1.00 × 10^−10^	5.00 × 10^−10^	1.00 × 10^−9^
2	10	0.01%	0.01%	0.01%	0.05%	0.01%	0.01%	0.04%	
	1	0.24%	0.22%	0.05%	0.02%	0.01%	0.01%	0.00%	0.02%
	0.1	28.39%	13.85%	2.49%	1.28%	0.28%	0.13%	0.03%	0.03%
	0.01		107.53%	33.23%	14.63%	2.85%	3.55%	0.60%	0.51%
20	10	0.04%	0.02%	0.01%	0.01%	0.01%	0.01%	0.01%	0.01%
	1	4.94%	2.52%	0.28%	0.22%	0.04%	0.03%	0.01%	0.01%
	0.1	185.66%	106.75%	20.30%	10.25%	2.90%	1.03%	0.21%	0.11%
	0.01				172.77%	20.94%	15.84%	5.53%	4.59%
200	10	0.65%	0.35%	0.11%	0.05%	0.01%	0.01%	0.00%	0.00%
	1	17.03%	11.79%	4.80%	2.52%	0.46%	0.23%	0.02%	0.03%
	0.1		657.51%	186.72%	110.41%	26.35%	10.46%	2.35%	1.03%
	0.01							29.39%	22.51%
2000	10	6.20%	3.38%	0.87%	0.50%	0.09%	0.06%	0.01%	0.01%
	1		70.66%	18.59%	12.95%	4.80%	2.50%	0.31%	0.34%
	0.1					235.31%	105.10%	20.83%	9.90%

**Table 9:** REM-2 no. 8 recombination chamber ionization current as a function of the X-ray exposure time. The detector was placed at a distance of 0.5 m from the beam axis. A PMMA phantom measuring 24 cm × 24 cm × 15 cm was placed in the beam. Comparison of the ionization current, calculated from the total charge accumulated during the exposure *I_calc_*, and the measured current, determined as the average of the points of the derivative of the charge during the exposure *I_meas_*.

NPLC	Ionization Current [nA]	Exposure Time [ms]												
		10	20	32	50	63	71	80	90	100	125	160	200	250
0.01	*I_calc_*	2.63	2.70	2.74	2.76	2.77	2.77	2.77	2.78	2.78				
	*I_meas_*	2.54	2.73	2.76	2.76	2.78	2.77	2.78	2.78	2.78				
	$\frac{{I_{meas}-I}_{calc}}{I_{calc}}$	−3.3%	1.3%	0.8%	0.1%	0.2%	0.0%	0.3%	−0.1%	0.0%				
0.1	*I_calc_*		2.73	2.76	2.77	2.78	2.78	2.78	2.78	2.79				
	*I_meas_*		2.71	2.75	2.78	2.79	2.79	2.79	2.79	2.79				
	$\frac{{I_{meas}-I}_{calc}}{I_{calc}}$		−0.9%	−0.1%	0.4%	0.3%	0.2%	0.2%	0.1%	0.1%				
1	*I_calc_*										2.79	2.78	2.79	2.79
	*I_meas_*										2.71	2.74	2.77	2.79
	$\frac{{I_{meas}-I}_{calc}}{I_{calc}}$										−2.8%	−1.6%	−0.5%	−0.1%

## Data Availability

The data supporting reported results are available on request by e-mail: michal.kuc@ncbj.gov.pl.
